# Composite Scaffolds from Gelatin and Bone Meal Powder for Tissue Engineering

**DOI:** 10.3390/bioengineering8110169

**Published:** 2021-11-01

**Authors:** Darlin Lantigua, Xinchen Wu, Sanika Suvarnapathaki, Michelle A. Nguyen, Gulden Camci-Unal

**Affiliations:** 1 Biomedical Engineering and Biotechnology Program, University of Massachusetts Lowell, One University Avenue, Lowell, MA 01854, USA; Darlin_Lantigua@student.uml.edu (D.L.); Xinchen_Wu@student.uml.edu (X.W.); Sanika_Suvarnapathaki@student.uml.edu (S.S.); 2 Department of Chemical Engineering, University of Massachusetts Lowell, One University Avenue, Lowell, MA 01854, USA; Michelle_Nguyen@student.uml.edu; 3 Department of Surgery, University of Massachusetts Medical School, 55 Lake Avenue North, Worcester, MA 01605, USA

**Keywords:** hydrogels, scaffolds, bone, photocrosslinking, tissue engineering

## Abstract

Bone tissue engineering offers versatile solutions to broaden clinical options for treating skeletal injuries. However, the variety of robust bone implants and substitutes remains largely uninvestigated. The advancements in hydrogel scaffolds composed of natural polymeric materials and osteoinductive microparticles have shown to be promising solutions in this field. In this study, gelatin methacrylate (GelMA) hydrogels containing bone meal powder (BP) particles were investigated for their osteoinductive capacity. As natural source of the bone mineral, we expect that BP improves the scaffold’s ability to induce mineralization. We characterized the physical properties of GelMA hydrogels containing various BP concentrations (0, 0.5, 5, and 50 mg/mL). The in vitro cellular studies revealed enhanced mechanical performance and the potential to promote the differentiation of pre-osteoblast cells. The in vivo studies demonstrated both promising biocompatibility and biodegradation properties. Overall, the biological and physical properties of this biomaterial is tunable based on BP concentration in GelMA scaffolds. The findings of this study offer a new composite scaffold for bone tissue engineering.

## 1. Introduction

Annually, 2.2 million bone grafts are performed worldwide for the treatment of non-union fractures or critical-size bone defects, creating a $2.5 billion industry [1,2]. Harvesting a patients’ own bones to replace damaged tissues, known as autografts, represent the gold standard grafting procedure [2]. However, autografts are frequently too expensive or are an inviable option. Additionally, they often require a secondary surgical procedure to obtain the grafting material. Moreover, the considerable time between tissue extraction and implantation can lead to donor site morbidity. Similarly, other alternatives such as allografts and distraction osteogenesis pose an increased risk of immunogenicity, disease transmission, and pin site infections. Despite major improvements, current medical procedures to treat bone-related injuries and bone loss still present major clinical risks and are not readily available patient options. As a result, these medical concerns related to conventional surgical procedures and bone substitutes have motivated bone tissue engineering strategies to overcome these clinical challenges [2,3,4,5].

Bone tissue engineering approaches, especially in orthopedic and dental areas, rely on both biocompatible materials and the innate ability of the human body to grow and remodel new bone. Specifically, these biomimetic materials have biological, chemical, and mechanical loading features that encourage osteoinductive or osteoconductive mechanisms [4,6,7]. Hydrogel-based scaffolds have therefore found a wide array of applications in this area since the physical and biological factors of a material are known to influence osteogenesis. In particular, the stiffness, degradation rate, porosity, pore size, and overall biological performance can be modulated in these scaffolds to meet the necessary requirements and cues that promote bone formation [2,8,9,10,11].

Naturally derived organic materials such as collagen and inorganic minerals such as hydroxyapatite (Ca_10_(PO_4_)_6_(OH)_2_) are increasingly utilized in engineered biocomposite hydrogels for bone tissue engineering applications [12,13,14,15]. As a main component of bone, hydroxyapatite works together with collagen type I to provide toughness and prevent bone ruptures while collagen serves in the regulation of cell attachment, proliferation, and differentiation [2]. Therefore, fabrication of hydrogels-based scaffolds with constituents of bone can yield promising results. When fabricated as a composite material, these polymeric materials and osteoconductive microparticles provide cues for cellular growth and differentiation of progenitor cells into osteogenic lineages [8]. These scaffolds often have improved mechanical properties, which is a limitation of many current hydrogels.

Herein, we report the fabrication of a novel composite scaffold composed of both gelatin and commercially obtained bone meal powder (BP) (KAL, Vitamins, Park City, UT, USA), a natural source of calcium and phosphorous. Gelatin is an analog of collagen and highly bioactive material with tunable biological, chemical, and physical properties. Gelatin can be modified with methacrylic anhydride to yield UV light photocrosslinkable gelatin methacrylate hydrogels (GelMA). GelMA comprises both natural cell binding sites and MMP-degradation sites (a desirable characteristic for in vivo implantation) [16,17]. The production of gelatin is low cost and the ability to modulate physical and biological materials properties provides a valuable resource for bone tissue engineering applications [12,18,19]. However, similar to other naturally derived hydrogels, GelMA hydrogels typically present weak mechanical properties [16]. Therefore, a strategy to address this shortcoming is to reinforce the material with microparticles to improve its overall mechanical strength.

Auto-, allo-, and xenografts, including bone fragments and powders, have been used for critical sized bone defects, among which autografts are considered to be the gold standard [4,20]. However, these grafts often require secondary surgeries, and have limitations including the risk of infection, biocompatibility, and osteoconductivity [20,21]. Furthermore, the fit of existing bone grafts is often incompatible with the site in need of bone repair. GelMA hydrogel is extremely pliable, and can be applied to defects of non-load bearing bones. The porous structure of the hydrogel is a favorable matrix to immobilize the micron size BP particles. The physical properties of the GelMA/BP hydrogels are highly tunable by varying the concentration of the BP. The GelMA/BP composites aim to provide mechanically improved and bioactive scaffolds. The outcomes of this work could replace traditional bone scaffolds by introducing highly flexible and tunable composites for the treatment of non-load bearing bone defects.

BP is a natural source that contains both calcium and phosphorous as its major components. These moieties support the material’s high osteoinductive and biocompatible properties [22]. Therefore, in this study, we hypothesized that a combination between BP and GelMA to form a composite material will result in a scaffold with enhanced mechanical properties and osteoinductive properties. The enhanced mechanical properties and bioactivity are critical parameters for bone regeneration [2,23]. As a novel scaffold, this biomaterial is expected to be suitable for promoting osteogenic differentiation and bone regeneration.

**Scheme 1:**
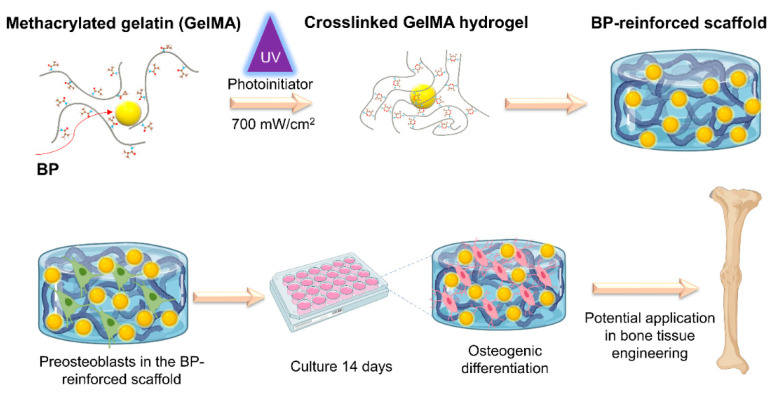
Composite scaffolds from gelatin and bone meal powder for bone tissue engineering. Modulating the concentration of the BP content within the GelMA matrix yields hydrogel products with tunable physical and enhanced mechanical properties while encouraging proliferation, differentiation, and mineralization of osteoblastic cells. The composite scaffolds demonstrate biocompatibility and biodegradability in vitro and in vivo.

Based on our experimental findings, our composite material presents tunable physical and biological characteristics. The in vitro cellular studies confirmed the osteoinductive properties of this material. Other material characterization tests demonstrated that BP enhances the mechanical strength of gelatin-based hydrogels. The in vivo results supported that this scaffold is biocompatible and biodegradable. These properties are controlled by modifying the BP concentration in GelMA (Scheme 1).

## 2. Materials and Methods

### 2.1. Materials

Bone meal powder was obtained from Kal Vitamins (Park City, UT, USA). Fetal bovine serum (FBS), trypsin-ethylenediaminetetraacetic acid, penicillin/streptomycin, Dulbecco’s phosphate buffered saline (DPBS), and Dulbecco’s modified eagle’s medium (DMEM) were obtained from Gibco (Thermo Fisher Scientific, Inc., Waltham, MA, USA). 2-hydroxy-1-[4-(hydroxyethoxy) phenyl]-2-methyl-1-propanone (Irgacure 2959) was obtained from BASF Corporation (Florham Park, NJ, USA). Microscope slides were supplied by VWR (Radnor, PA, USA). Alamar Blue reagent was purchased from Invitrogen (Grand Island, NY, USA). Sodium hydroxide, Alizarin Red S and Methacrylic anhydride were obtained from Sigma-Aldrich (St. Louis, MO, USA). Gelatin from porcine skin and dulbecco’s phosphate buffer saline (DPBS) (modified without calcium chloride and magnesium chloride) were obtained from Sigma-Aldrich (St. Louis, MO, USA). MC3T3-E1 pre-osteoblast cells (ATCC, Manassas, VA, USA). All reagents were used as received without further changes.

### 2.2. Synthesis of GelMA

The synthesis of GelMA was performed by combining 10 g of the gelatin component with 100 mL of DPBS at 50 °C [9]. Then, 8 mL of methacrylic anhydride was added to the solution and mixed for a reaction time of four hours. The reaction was quenched with 300 mL of DPBS to stop the methacrylation reaction. The resulting solution was dialyzed for 1 week and then freeze-dried for seven days after overnight freezing at −80 °C. The lyophilization resulted in a solid product that was then kept at −20 °C until use.

### 2.3. Production of GelMA/BP Hydrogels

To prepare GelMA hydrogels with different BP concentrations, the prepolymer precursor was prepared by mixing 0.5% (*w*/*v*) photoinitiator (PI) in DPBS at room temperature for a final GelMA concentration of 5% (*w*/*v*). The GelMA prepolymer solution was then mixed with different BP concentrations (0.5, 5, and 50 mg/mL) which we abbreviate as BP 0.5 mg/mL, BP 5 mg/mL and BP 50 mg/mL, respectively. As a control, hydrogels composed of only 5% (*w*/*v*) GelMA were also prepared. The hydrogels were prepared by pipetting 100 µL of polymer solutions between two glass slides that were set to 1 mm apart. Then, the precursor solution was exposed to UV light at a distance of 10 cm and 4 mW/cm^2^ intensity (Omnicure S2000, EXFO Photonic Solutions Inc., Mississauga, ON, Canada). Following, the crosslinked hydrogels disks were rinsed in DPBS to remove unreacted prepolymer material. The samples were stored in DPBS until use. This fabrication technique for GelMA/BP hydrogels was used in swelling, degradation, and mechanical testing. 

### 2.4. Swelling Behavior of GelMA/BP Hydrogels 

The swelling test was performed by submerging the hydrogel discs in a petri dish with 1 mL of DPBS for 24 h to reach equilibrium swelling. After equilibrium swelling, the gel discs were removed from DPBS and a kimwipe was used to remove excess liquid. The wet weight was determined before placing the gels into pre-weighed eppendorf tubes. The samples were stored at −80 °C overnight and then lyophilized. The dry weight of the gels was measured, and the swelling ratio was determined by dividing wet weight by dry weight of the gels. For this study, four replicates were used for each GelMA/BP composite hydrogel composition. 

### 2.5. Degradation of GelMA/BP Hydrogels

The preparation of hydrogels for the degradation study followed a similar process described in Section 2.3. After the gels were crosslinked, the excess unreacted polymer was removed, and each composite hydrogel disk was individually placed into an Eppendorf tube. The samples were frozen for 48 h at −80 °C before lyophilization. The dried hydrogels were then weighed out to obtain the initial dry weight. Following, 1 mL of DPBS was added to each Eppendorf tube to allow gels to reswell for 24 h. After 24 h, the DPBS was removed and 1 mL of collagenase IV (0.5 U/mL) in DPBS was added to each tube. The hydrogels were then placed in an incubator at 37 °C under shaking conditions at 130 rpm. The enzyme was removed at different time points (4, 8, 12, 18, and 24 h) and the composite hydrogel disks were then rinsed with DPBS to remove the excess enzyme still presented in the samples. After each time point, the samples were lyophilized to obtain the remaining dried weight of each hydrogel. Percent mass remaining was determined by dividing the hydrogel weight after degradation by the initial dry weight. The results were converted into percent values. In this study, four replicates were used for each GelMA/BP composite hydrogel composition.

### 2.6. Mechanical Testing 

To prepare samples, fabricated hydrogels were stamped into a disk shape with a diameter of 8 mm and a thickness of 1 mm. The mechanical strengths of the hydrogels were evaluated using the Dynamic Mechanical Analyzer (DMA) TA Instruments Q800 (New Castle, DE 19720, USA) under the stress/strain mode. The test was conducted on a calibrated DMA instrument with a compliance of ≤1.3, under a control force of 0.1 N/min until a maximum force of 2 N. The Poisson ratio was set to 0.5. The test was stopped at 10 min to collect data points for determining the compressive modulus of the hydrogels. To calculate the strain percentage, the percent difference in position of the hydrogel at each point was used. The stress of the materials was calculated in kPa by dividing the static force (N) at each point by the area of the surface of the hydrogel disc (mm^2^) and then multiplying the result by 1000. In this study, four replicates were used for each GelMA/BP composite hydrogel composition.

### 2.7. Scanning Electron Microscopy (SEM) Imaging

The hydrogels were prepared according to the method used for swelling and degradation assays. The Pristine GelMA, BP 50 mg/mL, and BP only were flash frozen and freeze dried. Subsequently these samples were hand broken and mounted on an aluminum stub with carbon tape. The samples were then sputter coated with gold. SEM images of the scaffold cross-sections were then obtained using a Field Emission Scanning Electron Microscope (JEOL JSM 7401F, Peabody, MA, USA). These SEM images were analyzed using NIH Image J 1.51 software. The SEM images show the pore structure and the particle distribution within the scaffolds. The SEM images reveal that the average size of the bone meal powder particles was about 10 μm. The average pore size of the BP 50 mg/mL scaffolds was 30 μm whereas the average pore size of the GelMA only scaffolds is about 5 μm.

### 2.8. Three-Dimensional (3D) Encapsulation of Preosteoblasts in GelMA/BP Composite Hydrogels 

The hydrogel prepolymer solution was prepared by mixing the polymer precursor (GelMA) in DPBS at a concentration of 5% (*w*/*v*) with 0.5% (*w*/*v*) photoinitiator (PI). The BP was added to the prepolymer solution at 0.5, 5, and 50 mg/mL concentrations. A 5% (*w*/*v*) GelMA hydrogel was used as a negative control. The solutions were vortexed for a few seconds to obtain a homogenous mixture and kept on a hot plate at 37 °C until UV crosslinking. The MC3T3-E1 pre-osteoblast cells used in this study were cultured in DMEM medium supplemented with 10% (*v*/*v*) FBS and 1% (*v*/*v*) penicillin/streptomycin. The cell culture was maintained in an incubator at 37 °C with 5% CO_2_ supplementation. For encapsulation, MC3T3-E1 cells were trypsin-treated to facilitate dissociation from the culture flasks. The cells (5 million cells/mL) were placed in a Falcon tube and centrifuged. The cell pellet was resuspended in the prepolymer solutions and pipetted up and down for homogenous cell distribution. To photocrosslink, 10 µL of the resulting cell/prepolymer solutions were placed between a prepared petri dish spacer (150 µm) and a glass slide. The resulting hydrogels were obtained after a four second exposure to UV light. After, the resulting hydrogels were rinsed with DPBS and cultured for 14 days in α-MEM media supplemented with 10% FBS and 1% (*v*/*v*) penicillin/streptomycin. The culture media was replenished every 2–3 days. The samples were evaluated for cellular metabolic activity, ALP activity, and osteogenic gene expression. In these in vitro studies, three replicates were used for each prepolymer solution. 

### 2.9. Alamar Blue Assay

The Alamar blue assay evaluated the metabolic activity of preosteoblasts that were 3D-encapsulated in composite GelMA/BP hydrogels. This assay was conducted on days 1, 4, 7, and 14 using the manufacturer’s protocol. The 3D-encapsulated preosteoblasts were incubated in 10% (*v*/*v*) Alamar blue solution for three hours. The incubator remained in normal cell culture conditions (37 °C and 5% CO_2_). The fluorescence values of the resulting solutions were obtained using a plate reader (Spectramax, San Jose, CA 95134, USA) at 560/590 nm (Ex/Em). 

### 2.10. Alkaline Phosphatase Activity

An alkaline phosphatase (ALP) assay was used to evaluate the intracellular ALP activity of the preosteoblast cells 3D-encapsulated in GelMA/BP hydrogels, which functions as a biomarker to indicate early osteogenic differentiation. The Alkaline Phosphatase Assay kit (Anaspec, Inc., Fremont, CA, USA) was used according to the manufacturer’s protocol after days 1, 4, 7, and 14 in culture. The preosteoblast cells were lysed with 600 μL of the lysing buffer solution provided with the commercial kit. The lysed cells were then centrifuged for 15 min at 10,000 G. The supernatant was removed and then added to a 24-well culture plate. This was followed by the addition of p-nitrophenyl phosphate disodium (PNPP) to each well. The 24-well culture plate was incubated for 1 h at 37 °C. After incubation, the stop solution was added to each well. Following, the absorbance of the solution was measured using a plate reader at a wavelength of 405 nm. 

### 2.11. RT-PCR for Osteogenic Gene Expression

The total RNA was isolated from the 3D-encapsulated preosteoblast GelMA/BP samples post 14 days in culture. A RNAqueous kit (Invitrogen) was used following the manufacturer’s protocol. Using Nanodrop 2000 (Waltham, MA, USA), the purity and quantity of total RNA in each sample was determined. Following the manufacturer’s protocols, the RT-qPCR Kit (verso One-Step), SYBR Green and Low ROX (Thermo-Fisher, Waltham, MA, USA) were utilized and run on the CFX Connect Real-Time System (Bio-Rad). Expression of housekeeping gene, GAPDH, was used to normalize the expression of targeted genes. The following forward and reverse PCR primers were used: BMP-7 (5′-TACATGGGAAACCTGGGTAAAG-3′ and 5′-GGTGACATTCTGTCGGGTAAA-3′), Osteocalcin (5′-TGTGTCCTCCTGGTTCATTTC-3′ and 5′-CTGTCTCCCTCATGTGTTGTC -3′), Osteonectin (5′-ATGAGGGCCTGGATCTTCTT-3 and 5′-GCTTCTGCTTCTGAGTCAGA-3′) and GAPDH (5′-CGCCCTGATCTGAGGTTAAAT-3′ and 5′-CGGAGCAACAGATGTGTGTA-3′). The expression of the targeted genes was used as an indication of differentiation of the preosteoblast cells. 

### 2.12. Mineral Staining

The calcium portion of the minerals in the GelMA/BP hydrogels was determined through alizarin red staining. The staining was performed in all composite GelMA/BP hydrogel conditions (BP 0.5, BP 5, and BP 50 mg/mL) devoid of cells. A 2% (*w*/*v*) alizarin red staining solution prepared in deionized (DI) water at pH 4.4 was used to stain all the hydrogel samples by incubation of the samples for 20 min in this solution. After staining, the scaffolds were washed four times with DI water until the washing solution became clear. The samples were imaged, and quantification of red color was determined using ImageJ software. In addition, the hydroxyapatite portion of the minerals was determined through a commercially available fluorescent staining kit (Osteoimage, Lonza) in the GelMA/BP composite scaffolds at different BP concentrations. The intensity of the green fluorescence represents the amount of the hydroxyapatite in the scaffolds. Osteoimage staining revealed an enhanced concentration of the hydroxyapatite mineral content in composite scaffolds containing BP microparticles. The quantification of the green fluorescence was carried out using the NIH ImageJ. 

### 2.13. In Vivo Subcutaneous Implantation and Histology Staining 

The GelMA/BP hydrogels were implanted in a rat model to assess biocompatibility and biodegradation in vivo. The in vivo implantation studies were performed in the animal research facility at the University of Massachusetts Lowell in compliance with the approved protocol and guidelines specified by Institutional Animal Care and Use Committee (IACUC) (17-05-07-Una). For this study, Wistar rats (100–150 g weight) obtained from Charles River Lab (Wilmington, MA, USA) were used for the subcutaneous implantation study. Prior to implantation, sterile 5% (*w*/*v*) GelMA hydrogels with a 50 mg/mL BP concentration and hydrogels made of 5% (*w*/*v*) pristine GelMA were prepared. In this study, three rats (*n* = 3) per the hydrogel conditions were used. Before surgery, rats were anesthetized using isoflurane. Under aseptic conditions, the dorsal incision areas were cleaned. A subcutaneous pocket was then generated on the right side of each incision. Then, sterile BP 50 mg/mL and 5% (*w*/*v*) pristine GelMA hydrogels were inserted into the subcutaneous pocket of the rats. Immediately post-implantation, the incisions were closed, and the animals were then put into recovery. The implantation sites were monitored for 14 days for signs of inflammation. After 14 days, the animals were sacrificed, and hydrogels were explanted along with surrounding tissues. To evaluate the explanted samples, they were first embedded in optimal cutting temperature (OCT) liquid at −80 °C and 10 µm thick samples were obtained through cryo-sectioning. The samples were then mounted on microscope slides and Hematoxylin/Eosin (H&E) staining was performed following standard protocols. 

### 2.14. Statistical Analysis

The statistical analyses were achieved by using GraphPad Prism (La Jolla, CA, U.S.A.). All the statistical data was determined by one-way and two-way ANOVA in combination with Bonferroni tests. In this work, the data were represented as an average ± standard deviation (* *p* < 0.05, ** *p* < 0.01, *** *p* < 0.001, and **** *p* < 0.0001).

## 3. Results

In this work, the physical and biological properties of composite hydrogels composed of 5% (*w*/*v*) methacrylated gelatin and BP at different concentrations (BP 0.5, BP 5 and BP 50 mg/mL in GelMA) were characterized in vitro and in vivo. The physical properties including swelling, degradation, and mechanical behavior were assessed for each hydrogel composition. In addition, the biological properties including the proliferation, differentiation, and biomineralization of preosteoblasts 3D-encapsulated within the composite GelMA/BP hydrogels were evaluated. The in vivo studies involved the subcutaneous implantation of the composite hydrogel material in a rat model. The in vivo studies revealed the biocompatibility and biodegradation properties of the scaffold. 

### 3.1. Swelling Behavior of the Composite Hydrogels

Hydrogels are hydrophilic materials capable of maintaining more than 90% water within their three-dimensional crosslinked structures. The swelling property of a hydrogel-based material is an important parameter influenced considerably by material porosities. Material swelling characteristics have been known to influence cellular infiltration in vivo. The swelling degree also has implications for the material’s ability to allow nutrients and waste products to diffuse within a hydrogel material [16,24].

**Figure 1:**
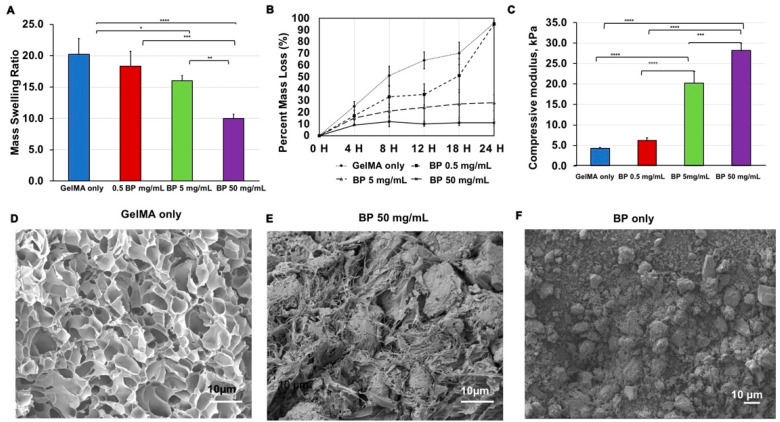
Characterization of the physical properties of GelMA/BP composite hydrogels. (**A**) Swelling analysis showed mass swelling ratios decreased with increased BP microparticle concentrations (Error bars: ±SD, * *p* < 0.05, ** *p* < 0.01, *** *p* < 0.001, and **** *p* < 0.0001). (**B**) Enzymatic degradation and (**C**) Compressive moduli analysis also revealed tunable behaviors based on the hydrogel composition. (Error bars: ±SD, *** *p* < 0.001 and **** *p* < 0.0001). Scanning Electron Microscopy (SEM) images of (**D**) GelMA only, (**E**) BP 50 mg/mL and (**F**) The BP only.

The swelling behavior of GelMA/BP hydrogels is shown to be tunable by changing the composition of BP within the composite hydrogel material (Figure 1). The addition of different BP concentrations influences the porosity of the GelMA/BP scaffolds, which also influences the swelling behavior of the GelMA/BP scaffolds. Larger pore sizes allow for a greater volume capacity within the scaffold’s polymer network, leading to increased swelling compared to scaffolds with smaller pore sizes. The addition of BP to the GelMA matrix decreases the porosity and allows lower water volume to infiltrate the scaffold’s polymer network, leading to less swelling as compared to the pristine GelMA scaffolds which have larger pore sizes. Therefore, scaffolds with a higher concentration of BP swell less compared to the scaffolds with no BP. Composite GelMA/BP hydrogels synthesized with a higher composition of BP showed a decrease in swelling ratio, when compared with hydrogels composed of GelMA-only or hydrogels containing lower amounts of BP (*p* < 0.0001) (Figure 1A). For example, 5% (*w*/*v*) GelMA showed the highest swelling ratio of 20.2 ± 2.5. In contrast, the BP 50 mg/mL hydrogel has significantly decreased swelling ratio of 10 ± 0.7. However, at lower concentration such as BP 0.5 mg/mL there was a minimal decrease in the swelling ratio (18.3 ± 3.4). This phenomenon is also observed in the BP 5 mg/mL hydrogels (16 ± 0.8). 

The incorporation of BP into the GelMA matrix decreases the hydrophilic GelMA component within these scaffolds [9,10,25]. Due to the reduced matrix hydrophilicity, there is less water retention within the GelMA/BP scaffolds and, as a result, they swell less compared to the GelMA scaffolds with no BP. 

Swelling ratios often decrease as a result of increased crosslinking time. In particular, these higher crosslinking times produce stronger interactions and bonding between hydrophilic polymeric chains of the hydrogel component of the scaffold. In this case, the addition of BP to the GelMA increased the crosslinking time required to obtain uniformly crosslinked scaffolds. Moreover, these scaffolds with different BP concentrations were crosslinked at different UV crosslinking times to ensure that the scaffolds were uniformly crosslinked for all BP concentrations.

Consequently, the network of hydrophilic chains permits a reduced capacity for water retention and swelling. Similarly, the spaces occupied by the BP component may have also reduced swelling in this study. By increasing the BP content, there is less of the GelMA component, which will in turn reduce the hydrophilic component of the hydrogel. The swelling behavior demonstrated a concentration dependency on the BP content in GelMA. Based on these results, we can modulate the swelling property by adjusting the BP content in GelMA scaffolds.

### 3.2. Degradation of the Composite Hydrogels

Material degradation is an important factor to consider for developing tissue engineered bone constructs, in part due to its influence over tissue ingrowth. Degradation of the scaffold material is necessary for cellular proliferation, ECM production and deposition, and new bone tissue formation. Ideally, engineered scaffolds should exhibit a controlled degradation rate while producing no cytotoxic byproducts during the degradation process. Therefore, hydrogels that biodegrade under physiological conditions are promising materials for bone regeneration [2].

The enzymatic degradation of composite GelMA/BP hydrogels by collagenase IV was conducted in vitro (Figure 1B). Under shaking conditions at 130 rpm and in a 37 °C environment, collagenase IV at 0.5 U/mL was shown to degrade the gelatin component of the composite hydrogels. Again, the results demonstrated that degradation is a tunable property of GelMA/BP hydrogels. Specifically, there was inverse relation between the BP concentration and degradation rate. For example, decreasing BP concentrations resulted in increasing degradation rates. At 24 h, the BP 50 mg/mL showed the lowest mass loss of 11%. Similarly, when the BP concentration was decreased to 5 mg/mL, the mass loss of 28% at this composition. At significantly lower BP concentrations, the degradation behavior was comparable to GelMA-only. In the BP 0.5 mg/mL, the hydrogel reached almost complete degradation at 24 h (95% mass loss). This mass loss percentage was almost equivalent to the 96% mass loss in the GelMA-only condition (96% mass loss). The collagenase enzyme used in the degradation experiments specifically only degrades the gelatin component of the hydrogels. Since the GelMA only condition has the highest amount of gelatin content, it shows the fastest degradation rate.

The incorporation of microparticles or nanoparticles within hydrogels results in composite hydrogels with improved mechanical properties [10,24]. Generally, the addition of particles results in stiffer hydrogels, which is a parameter that has been shown to affect enzymatic degradation behavior [26]. The collagenase enzyme used for the accelerated enzymatic degradation experiment, solely degrades the gelatin component of the GelMA/BP scaffold. Therefore, in scaffolds with higher BP concentrations within same volume of GelMA, there is less gelatin content present which consequently leads to lower % mass loss during degradation. Therefore, the degradation behavior is predictable based on the BP concentration in the hydrogel scaffold. 

### 3.3. Mechanical Properties of the Composite Hydrogels

The mechanical properties of a hydrogel-based scaffold are proportional to the crosslinking density, stiffness, and porosity of the scaffold [7,27]. Mechanical characteristics of a scaffold provides cues that influences cell spreading, proliferation, and differentiation within a 3D construct. Moreover, the stiffness of a hydrogel-based scaffold can also affect osteogenic differentiation and mineralization [2,16].

The mechanical testing results support that changes of the mechanical properties in GelMA/BP hydrogels are as a result of changes in the BP concentration in the hydrogels. The compressive modulus (kPa) increased as BP content increased in the hydrogels (Figure 1C). As demonstrated, there is a significant difference between the compressive moduli of the BP 50 mg/mL and GelMA-only hydrogel (28.2 ± 1.8 kPa and 4.3 ± 0.2 kPa, respectively). The BP concentration between this range influences the overall mechanical strength accordingly. In the BP 5 mg/mL and BP 0.5 mg/mL conditions, the compressive moduli were 20.2 ± 2.9 kPa and 6.2 ± 0.7 kPa, respectively.

As discussed, the mechanical strength of hydrogels composed of natural polymers are commonly improved with microparticle reinforcement. This behavior coincides with the results shown in this study. The improved and facile modulation of mechanical properties can support this scaffold as a viable solution for non-load-bearing applications such as cranial defects [3]. 

### 3.4. Scanning Electron Microscopy (SEM) Imaging

The microstructure of the GelMA/BP hydrogel scaffolds was characterized using SEM imagining. The hydrogel scaffolds were prepared according to the methods described in the previous section. The samples were then flash frozen by submerging in liquid nitrogen and subsequently freeze dried and then gold coating was performed on the freeze-dried samples. Field emission scanning electron microscope (JEOL JSM 7401F, Peabody, MA, USA) was used to perform the cross-sectional imaging of the scaffolds. The pore structure and BP particle distribution within the scaffolds can then be visualized (Figure 1D–F). The SEM images revealed the porous interconnected structure of the pristine GelMA and BP 50 mg/mL scaffolds. The SEM images were imported in Image J version 1.51 and analyzed by fitting ellipses within the imaged pores to calculate the average pore size. In these analyses, three replicate images were analyzed per scaffold condition. The diameter values of 300 pores per sample image were measured on an average, and the average sizes and standard deviation values for the pores and BP particles were calculated. The imaging confirmed that the BP particles have an average particle size of 10 μm ± 3.1 μm, whereas the pristine GelMA scaffolds have an average pore size of 5 μm ± 6.1 μm. The BP particles homogeneously integrate within the GelMA matrix and form porous scaffolds with homogeneous mechanical reinforcement. Additionally, the porous structure of the scaffolds offers a biomimetic synthetic extracellular matrix in which cells can be grown and differentiated.

### 3.5. Mineral Staining of GelMA/BP Scaffolds

**Figure 2:**
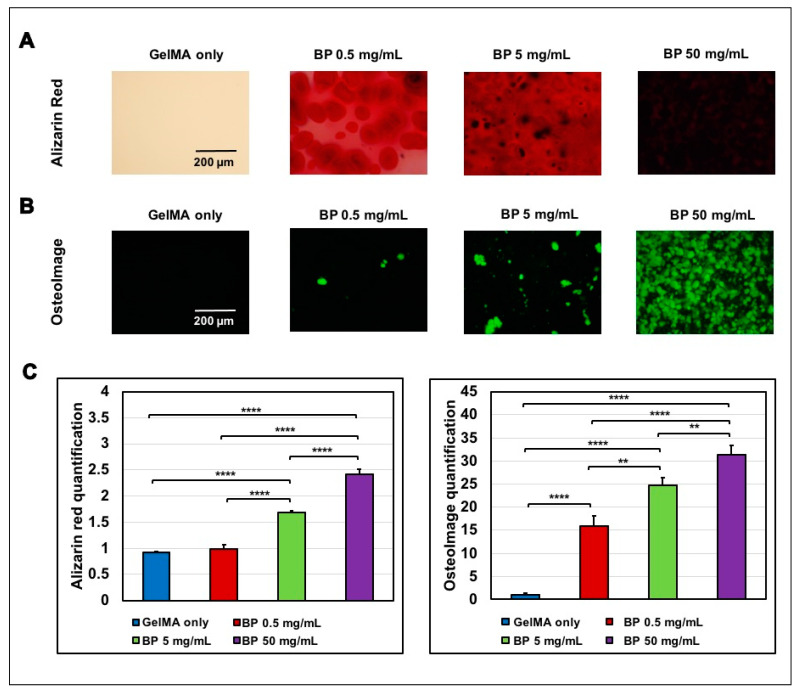
Evaluation of the calcium and hydroxyapatite components in the GelMA/BP composite hydrogels. The alizarin red images were taken by staining scaffolds without cells so that the alizarin red dye stains calcium content due to the presence of BP within the scaffolds. (**A**) Alizarin Red staining of calcium-rich portions (Scale bar: 200µm). (**B**) Osteoimage staining of hydroxyapatite portion in green (Scale bar: 200µm). (**C**) Quantification of the red color intensity in Alizarin Red staining (Error bars: ±SD, ** *p* < 0.01, **** *p* < 0.0001) and quantification of the green fluorescence in Osteoimage staining.

The calcium rich and hydroxyapatite rich zone within the GelMA/BP scaffolds were imaged using Alizarin red staining and osteoimage staining respectively (Figure 2). The quantification of alizarin red and osteoimage staining indicate that BP 50 mg/mL scaffolds have significantly higher calcium and hydroxyapatite contents compared to the GelMA only scaffolds.

### 3.6. Proliferation of 3D Encapsulated Preosteoblasts in Composite Hydrogels

**Figure 3:**
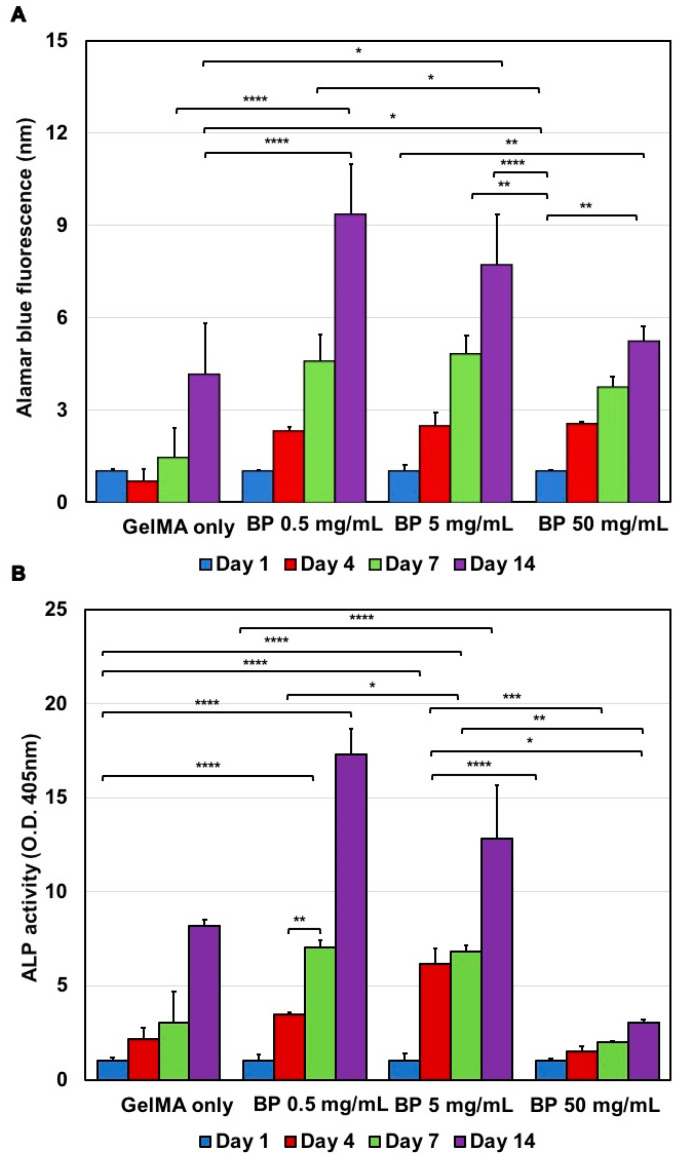
Cellular responses to various GelMA/BP composite hydrogels. (**A**) Metabolic activity of the 3D-encapsulated preosteoblasts from day 1 to day 14 with enhanced effects shown at high BP concentrations. Fluorescence values of alamar blue assay at 560/590 nm and normalized to day zero (Ex/Em); error bars: ± SD, * *p* < 0.05, ** *p* < 0.01 and **** *p* < 0.0001). (**B**) Alkaline phosphatase (ALP) activity of 3D-encapsulated preosteoblasts from day 1 to day 14 reflected behavior shown in osteogenic differentiation. ALP activity values represented as absorbance values at 450 nm and normalized to day zero values (Error bars: ± SD, * *p* < 0.05, ** *p* < 0.01, *** *p* < 0.001, and **** *p* < 0.0001).

The purpose of the 3D encapsulation of preosteoblasts is to evaluate the cell attachment, proliferation, and differentiation in GelMA/BP in vitro. These results are necessary to assess the material biocompatibility and suitability as a tissue engineered construct [24,28]. For this study, we used the Alamar Blue assay to evaluate the proliferation of pre-osteoblast cells within these composite hydrogels. The proliferation of the cells was evaluated in all hydrogel conditions (BP 0, 0.5, 5, and 50 mg/mL) at days 1, 4, 7, and 14 (Figure 3). In Figure 3A, there was a substantial increase in proliferation across all hydrogel conditions from day 1 to 14. The proliferation of cells can be correlated to the viability of the cells during the in vitro encapsulation study. 

The results demonstrated a higher increase in proliferation of the cells encapsulated in the all the BP compositions (BP 0.5, 5 and 50 mg/mL) in comparison to the GelMA-only group. Specifically, the 0.5 mg/mL GelMA/BP condition showed the highest proliferation rate on day 14 among all groups. The result suggests at this condition a superior proliferation rate was achieved. Conversely, at BP 50 mg/mL, it was observed that the metabolic activity at day 14 was decreased for composite hydrogels. In particular, the 50 mg/mL BP composition showed the lowest metabolic activity at day 14. The decrease in the proliferation for this group can be a result of cell differentiation. Literature studies have shown that cells proliferate to confluence and then differentiate [29]. Thus, decreasing proliferation rates possibly indicate an increasing differentiation which is consistent with the ALP activity and gene expression results as provided in the next section. This in vitro proliferation study demonstrates that within a certain range, the incorporation of BP within GelMA hydrogels enhances the proliferation over time. Overall, this indicates that the scaffold is cytocompatible and can be modified to modulated cellular responses by BP doping. 

### 3.7. Alkaline Phosphatase (ALP) Activity

The biochemical signals in the ECM play a crucial role in the regulation of cellular behavior and their processes. Therefore, it is advantageous to generate scaffolds that can be modified to modulate biological responses and guide cells to proliferate and differentiate [22]. In this study, we monitored the effect of BP concentration on the differentiation behavior of preosteoblast by measuring their intracellular ALP activity. ALP is commonly known as an early differentiation marker in osteoblastic cells and its activity was monitored over a time period of 14 days (Figure 3B) [30].

The results demonstrated a higher ALP activity in composite hydrogels synthesized with a 0.5 or 5 mg/mL BP composition during the 14-day in vitro study. In contrast, at the highest concentration (BP 50 mg/mL), the ALP activity shown by preosteoblasts was the lowest. In addition, 5% (*w*/*v*) GelMA-only also demonstrated a comparable ALP activity similar to the 0.5 and 5 mg/mL BP groups. Interestingly, there was the highest ALP activity in the BP 5 mg/mL group at day four. Then, at later time points, the BP 0.5 mg/mL group showed the highest ALP activity among the groups tested (days 7 and 14). These results collectively suggest that BP incorporated within the scaffolds affect the scaffold properties such as mechanical strength, and porosity which consequently can improve and control osteogenic differentiation over time. However, the optimal BP concentration to achieve maximized osteoinduction is potentially variable.

This study also observed a decrease in ALP activity of preosteoblasts over time in scaffolds with high BP content. A progressive decrease in ALP activity was observed in the BP 5 and BP 50 mg/mL groups at day 14, where the BP 50 mg/mL group showed the most prominent ALP decrease. The decreased ALP activity in these scaffolds with high BP indicated successful cell differentiation into an osteogenic lineage. Since ALP is an early bone differentiation marker, and ALP expression increases until osteogenic differentiation progresses [31,32]. Therefore, the ALP activity in the 3D-encapsulated preosteoblasts reflected the cellular responses involved in osteogenic differentiation. Most importantly, these results were achievable without the incorporation of other osteogenic factors such as bone morphogenic protein 2 (BMP-2). Overall, modulating the BP content in the GelMA is a feasible method to fine-tune the mineralization and osteoinductive effect of composite scaffolds to support the necessary cues to guide osteogenic cell differentiation.

### 3.8. Osteogenic Gene Expression for Preosteoblasts

**Figure 4:**
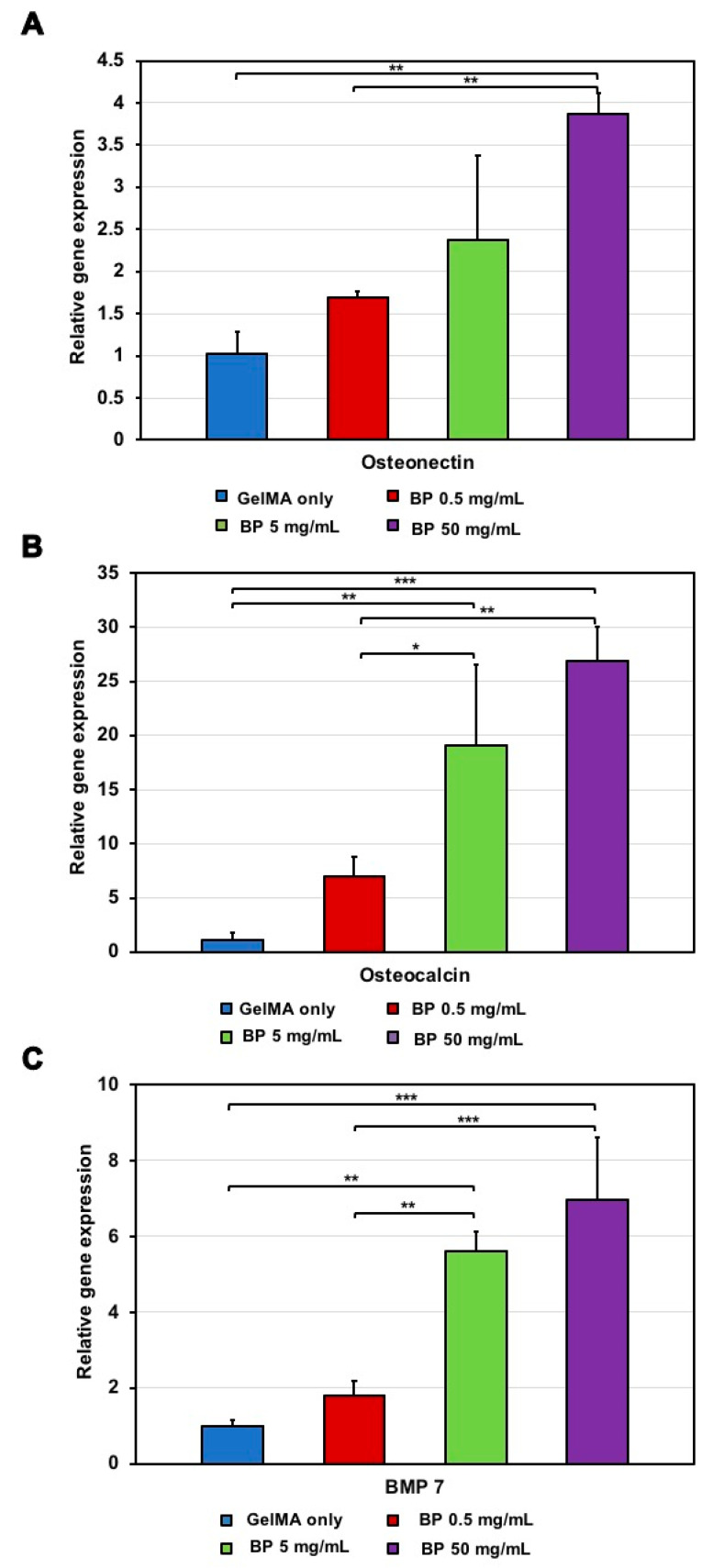
RT-PCR for osteogenic gene expression for preosteoblasts in GelMA/BP hydrogels. (**A**) Osteonectin expression upregulated at high BP concentrations (Error bars: ±SD, ** *p* < 0.01). (**B**) Osteocalcin expression upregulated at high BP concentrations (Error bars: ±SD, * *p* < 0.05, ** *p* < 0.01, and *** *p* < 0.001). (**C**) BMP 7 expression increased at high BP concentrations (Error bars: ±SD, ** *p* < 0.01 and *** *p* < 0.001). Results were normalized to the GAPDH gene.

The osteoinductivity of preosteoblast cells 3D-encapsulated in GelMA/BP hydrogels was validated by RT-qPCR. The bone differentiation markers studied included the bone morphogenic protein 7 (BMP-7), osteocalcin (OCN), and osteonectin (ON). These biomarkers were examined for their expression at day 14 (Figure 4). Regulation of these markers is critical as they are involved in osteogenesis and are late markers for osteogenic differentiation. For example, BMP-7 plays an important role in endochondral ossification and bone development [33]. Similarly, OCN has a regulatory role in the maintenance of bone mineralization, and ON modulates mineralization by liking minerals to type I collagen fibers [34]. We utilized the regulation of these markers as indications of the differentiation of osteoblastic cells.

The RT-qPCR results demonstrated a correlation between the BP composition within the hydrogels and the upregulation of all the markers. In particular, increasing the BP composition resulted in a higher biomarker expression. For instance, the BMP-7 regulation in the BP 50 mg/mL composition was significantly higher than in the 0.5, 5 mg/mL BP and GelMA-only compositions. Likewise, the OCN and ON expression levels in both the BP 5 and BP 50 mg/mL compositions are considerably higher in comparison to the BP 0.5 mg/mL and GelMA-only scaffolds. Specifically, the BP 50 mg/mL composition exhibited exceptional biological performance with high gene upregulation of OCN, ON and BMP-7, suggesting enhanced effects on the osteoblastic differentiation with respect to the other scaffolds. The results revealed that the incorporation of the BP content supports and promotes osteoblastic differentiation. Overall, the expression of these differentiation biomarkers can be controlled by modification of the BP content in these constructs.

The evaluation of the physical properties of the GelMA/BP scaffolds reveal an interesting correlation between the swelling behavior of the scaffolds and other physical and biological properties such as porosity, mechanical strength, and biological effect on the cells encapsulated within the scaffolds. For instance, the porosity of the scaffolds is impacted by the addition of different BP concentrations. The addition of BP decreases the average pore size which, in turn, allows less space for water to enter the polymer matrix. This outcome decreases the swelling ratio of the scaffolds. The rate and extent of swelling impacts the mechanical stiffness of the scaffolds, which guides the cellular microenvironment and the mechanical cues to the cells. Thus, consequently modulating the cellular viability, proliferation, and osteogenic differentiation experienced by the cells microencapsulated within the composite scaffolds. The variation in cellular responses was investigated through the in vitro experiments in this work.

### 3.9. Subcutaneous Implantation of the Composite Hydrogels and Histology

**Figure 5:**
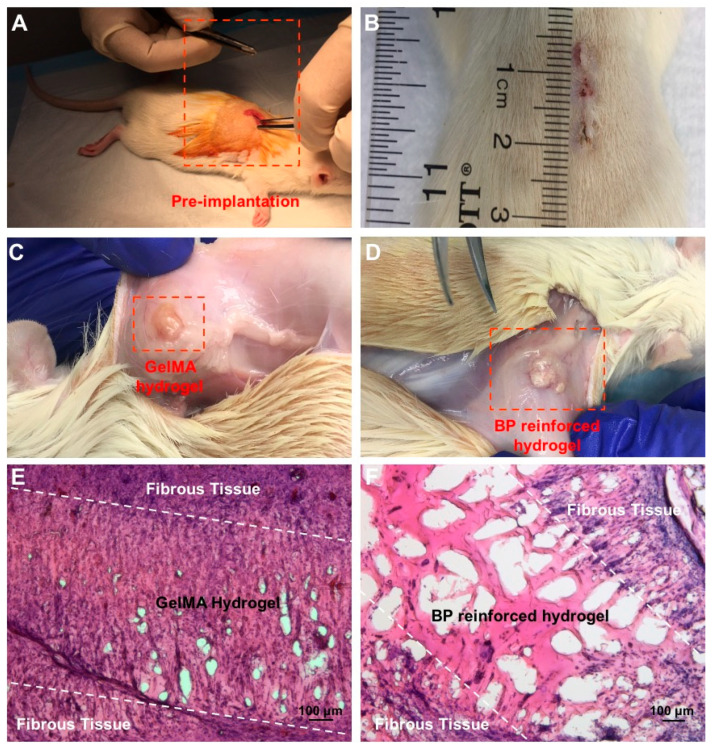
In vivo evaluation of subcutaneous implantation of GelMA/BP hydrogels. (**A**) Incision and subcutaneous pocket on the dorsal skin of the rat model pre-implantation. (**B**) Complete closure and healing of the incision 14 days post-implantation. (**C**) GelMA-only and (**D**) BP 50 mg/mL implants 14 days post-implantation. (**E**) H&E staining for the explanted tissue from 5% (*w*/*v*) GelMA-only and (**F**) BP 50 mg/mL implants 14 days post-implantation. Fibrous tissue, tissue infiltration, and minimal inflammation present within the degraded regions of both composite and control hydrogel scaffolds. Improved integration shown in BP 50 mg/mL with no presence of necrosis.

Biocompatibility and biodegradation properties were assessed in a rat model to further understand how the material integrates in vivo. In particular, there were two conditions tested in these models, namely BP 50 mg/mL and GelMA-only compositions. These hydrogels were subcutaneously implanted under the dorsal skin of a rat model. Standard Hematoxylin/Eosin (H&E) staining were conducted on the explanted materials and their surrounding tissues 14 days post-implantation (Figure 5). The histology results in both groups were compared. Figure 5 represents the subcutaneous pre-implantation pocket and post-implantation results obtained for the implanted GelMA/BP composite hydrogel.

The immune response shown in this study has potential clinical implications. Specifically, the immune response can be observed in a number of phases including the proliferation phase, fibrous tissue encapsulation, and macrophage infiltration into the implant [3,35]. The H&E staining revealed significant host cell infiltration and tissue in growth in the GelMA/BP material than in the control. However, both conditions presented minimal inflammatory responses to the subcutaneous implantations of the materials. The local inflammations were resolved by day 14 as demonstrated in the H&E staining (Figure 5E,F). Similar results have been shown by other literature studies [36]. The histological staining also revealed the presence of fibrotic tissue encapsulation in both GelMA/BP and control at day 14, which indicated healing responses post-implantation [37]. The tissue infiltration was more conspicuous in degraded regions in the GelMA/BP group than in the control. There was no presence of necrosis in the explanted tissues in none of the experimental groups.

In addition, the H&E staining also demonstrated a significant enhancement in the physiological degradation capacity in the GelMA/BP condition than in the GelMA-only control. The in vivo results reflected the degradation behavior in vitro. The in vivo degradation suggests that the incorporation of the BP microparticle improves the in vivo biodegradability. Moreover, the explanted material can degrade to allow for tissue growth and remodeling. The in vitro and in vivo results validate the biocompatibility and biodegradation properties of the composite scaffolds. As shown in previous studies, these features are advantageous for a diverse range of disease modeling and therapeutic applications in tissue engineering [38].

## 4. Conclusions

In this work, we demonstrate that the photocrosslinkable and osteoinductive GelMA/BP hydrogels are promising tissue constructs for bone tissue engineering. The fabrication method of this study reveals the spatial control over the biomaterial properties. Specifically, through adjusting the BP content, both the physical and biological features of this constructs can be controlled. The combination of BP with gelatin methacrylate resulted in the synthesis of a biologically active scaffold. The addition of the BP microparticles enhances mechanical properties while encouraging proliferation, differentiation, and mineralization of osteoblastic cells. The results of mechanical analysis revealed that while incorporation of BP into GelMA improves the osteoinductivity and mechanical strength of the scaffolds, the maximum mechanical strength achieved is lower compared to the native bone. The GelMA/BP scaffolds are highly pliable and easy to manipulate to fit irregular defect shapes. Therefore, these results suggest that the GelMA/BP scaffolds could be useful for non-load bearing applications in bone tissue engineering. The increased mechanical properties of the hydrogel-based scaffolds provide better osteogenic differentiation stimulation for pre-osteoblasts [39]. The composite scaffolds also demonstrate biocompatibility and biodegradability both in vitro and in vivo. The in vitro degradation behavior occurred in physiologically relevant conditions and was predictable based on the BP content in the hydrogel. In the future, this property can be manipulated in studies that could involve the GelMA/BP vehicle for the delivery of small molecules such as therapeutics and growth factors. The in vivo results revealed proper integration with the host and supported tissue ingrowth without presence of necrosis and significant inflammation. Our findings show that the composite scaffolds have promising osteoinductive capabilities that could possibly be suitable for bone regeneration and repair. The GelMA/BP hydrogel properties can be tailored to myriad of applications involving mineralized tissues. This biocomposite is also suitable to treating injuries involved in the cranial and maxillofacial bones. Given these robust material properties, GelMA/BP is demonstrated to be a novel and advanced tissue scaffold.

## Data Availability

Not applicable.
